# State of the Art and Future Prospects of Virtual and Augmented Reality in Veterinary Medicine: A Systematic Review

**DOI:** 10.3390/ani12243517

**Published:** 2022-12-13

**Authors:** Masoud Aghapour, Barbara Bockstahler

**Affiliations:** Section of Physical Therapy, Small Animal Surgery, Department for Companion Animals and Horses, University of Veterinary Medicine, 1210 Vienna, Austria

**Keywords:** virtual reality, augmented reality, mixed reality, simulation, veterinary medicine

## Abstract

**Simple Summary:**

Virtual reality and augmented reality are rapidly progressing technologies in different fields, such as the gaming and entertainment industries, military training, professional simulation, education, and medicine. Researchers have evaluated the use of these technologies in human and veterinary medicine. Our goal in this systematic review was to evaluate the articles that used virtual or augmented reality in veterinary medicine, as well as medical articles with animal trials and report published protocols and results. The studies we included in our review reported new diagnostic/therapeutic methods, as well as new educational possibilities for the use of virtual or augmented reality in human or veterinary medicine. These articles mostly focused on assessing different methods, tools (hardware and software), and workflow, in addition to troubleshooting the reported protocols. According to the included articles, the application of these technologies can increase the scientific output of students and residents, reduce training costs, and promote ethical standards. However, we consider the absence of standard protocols for the application of these tools, their time-consuming nature, and their prices as study limitations.

**Abstract:**

Virtual reality and augmented reality are new but rapidly expanding topics in medicine. In virtual reality, users are immersed in a three-dimensional environment, whereas in augmented reality, computer-generated images are superimposed on the real world. Despite advances in human medicine, the number of published articles in veterinary medicine is low. These cutting-edge technologies can be used in combination with existing methods in veterinary medicine to achieve diagnostic/therapeutic and educational goals. The purpose of our review was to evaluate studies for their use of virtual reality and augmented reality in veterinary medicine, as well as human medicine with animal trials, to report results and the state of the art. We collected all of the articles we included in our review by screening the Scopus, PubMed, and Web of Science databases. Of the 24 included studies, 11 and 13 articles belonged to virtual reality and augmented reality, respectively. Based on these articles, we determined that using these technologies has a positive impact on the scientific output of students and residents, can reduce training costs, and can be used in training/educational programs. Furthermore, using these tools can promote ethical standards. We reported the absence of standard operation protocols and equipment costs as study limitations.

## 1. Introduction

Veterinary students must learn a wide variety of clinical skills in a short amount of time, and the majority of these skills must be applied from the first day on duty [1,2,3]. Many of these students experience negative emotions during their academic life, such as stress, excitation, or lack of self-confidence [4]. Reassessing and improving educational methods by using new technologies may not only promote veterinary students’ clinical skills but also increase the self-confidence of postgraduate veterinarians, allowing them to more efficiently contribute to practical work [3]. Recently, educational programs have transitioned from working on real animals, using invasive methods, to working on artificial models or simulators, with non-invasive methods, to comply with ethical standards in veterinary science, namely, the 3Rs principle (replacement, reduction, and refinement) [3,5]. However, the COVID-19 pandemic underlined the need for new e-learning methods, with it becoming increasingly evident that healthcare education should incorporate new digital technologies, such as artificial intelligence (AI), virtual reality (VR), augmented reality (AR), machine learning, etc. [6,7].

VR and AR are among the newest and most rapidly developing technologies used in medical science. The concept of virtual reality refers to a simulation that immerses the user in a three-dimensional (3D) environment. Virtual environments are generated by computers, and users interact with them through interactive devices, such as headsets, glasses, controllers, etc. Virtual reality simulations can be classified into three categories, namely non-, semi-, and full-immersive simulations [8].

Non-immersive VR provides a computerized environment that relies on a computer or a gaming console, monitor, and controllers. While using this technology, users remain aware of their real environment. Video games are examples of non-immersive VR. 

Semi-immersive VR offers a partially virtual environment, giving the user the feeling of being in a virtual environment while remaining aware of their physical surroundings. This kind of VR is often used in educational programs or in practicing professional skills, including flight or military training simulators. Semi-immersive VR allows users to navigate a 3D virtual environment using a high-resolution screen or powerful projectors and simulators that partially mimic real-world mechanisms. 

Full-immersive VR presents the most realistic simulation of a virtual environment, and is accompanied by sound and vision that replaces users’ real surroundings, allowing them to fully engage with the simulated virtual world. For full-immersive VR, high-resolution headsets or VR glasses are required, which are called a head-mounted display (HMD). They provide a wide and stereoscopic 3D view by splitting the display between two eyes, which is accompanied by input tracking that creates an immersive and realistic experience for users. This VR category is commonly used in the gaming and entertainment industries; however, it is now also being used in other sectors, such as in education and professional training [9,10].

AR is a technology that provides an interactive environment by combining computer-generated images and the users’ real world; thus, the real and virtual worlds are superimposed in this technology. In addition to visual and auditory information, AR can provide further computer-generated perceptual information, such as haptic and olfactory perception [8]. As an alternative to AR, mixed reality (MR) was utilized in some articles, which provides an environment by combining physical and digital elements and allowing for interaction between them.

The use of VR/AR is not limited to the gaming and entertainment industries—different types of VR or AR can be used in different fields, such as in education [11,12], military and sports training [13], architectural projects [14], etc. In human medicine, the use of AR/VR has also increased during the past decade, and different studies have investigated the effectiveness of these methods in different medical specialties, such as neurosurgery [15,16,17,18,19,20,21], surgery [22,23,24], ophthalmology [6,25], orthopedics [26,27], physical medicine and rehabilitation [28,29], urology [30], etc. However, despite advances in human medicine, VR/AR has been overlooked in the veterinary field; therefore, there is great capacity in veterinary medicine to use these technologies for educational, diagnostic, and therapeutic purposes.

In our systematic review, we aimed to investigate all published articles in veterinary medicine that used VR or AR technologies, in addition to the medical literature with a focus on animal trials. Our first goal in this systematic review was to evaluate the methods used in the literature, including their outcomes, and report the state of the art. Our second goal was to illustrate the potential use for VR/AR technologies in veterinary medicine, with a focus on clinical science and higher education.

## 2. Materials and Methods

We carried out our study according to the PRISMA guidelines reported by Moher et al. for reporting systematic reviews and meta-analyses [31].

### 2.1. Data Sources

We performed literature research on 14 July 2022 and collected articles by screening the PubMed, Web of Science, and Scopus databases.

### 2.2. Search Strategy

The articles in this study were researched and selected by M.A. Our Scopus search for the term ‘virtual reality OR augmented reality OR mixed reality’ within article titles yielded 45,046 articles. Our search for the term ‘veterinary medicine OR animal OR canine OR dog OR feline OR cat OR pet OR equine OR horse OR ruminant OR cattle OR cow OR bovine OR sheep OR ovine OR swine OR pig’ within article titles yielded 1,112,734 articles. We combined these search results by using the combine queries tool (‘virtual reality OR augmented reality OR mixed reality’ AND ‘veterinary medicine OR animal OR canine OR dog OR feline OR cat OR pet OR equine OR horse OR ruminant OR cattle OR cow OR bovine OR sheep OR ovine OR swine OR pig’) and reduced the number of articles to 121. We performed the same procedures for the PubMed and Web of Science databases. We extracted 32 and 45 articles from the PubMed and Web of Science databases, respectively. Furthermore, we included three articles in our study from literature references and other sources.

### 2.3. Inclusion Criteria

**Figure 1:**
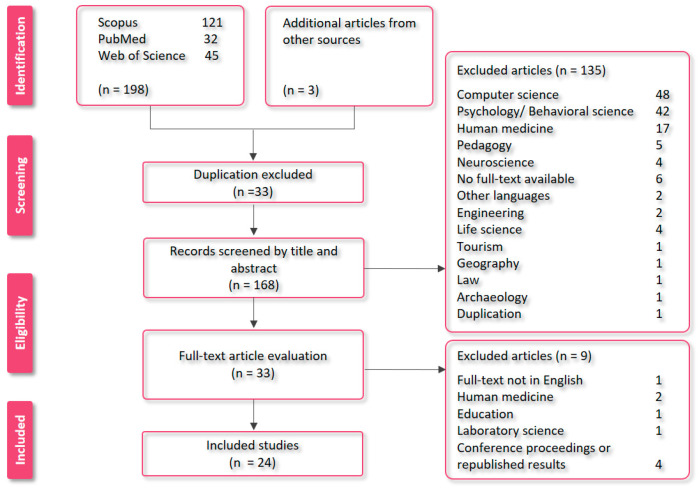
PRISMA flow diagram illustrating literature research, exclusion process, and number of included studies in systematic review.

All studies included in our systematic review had to be published in full-text and the English language. After combining our obtained articles (n = 201) from the three databases, we excluded duplications (n = 33) and assessed the remaining articles (n = 168) according to their titles and abstracts. We excluded all unrelated articles from the study (n = 135). The excluded articles mostly related to computer science, psychology, or behavioral science, and medical articles without animal trials. Figure 1 shows our search strategy and list of excluded articles.

### 2.4. Study Selection

We performed a final assessment of the remaining articles (n = 33) by reading the full text of the articles. As mentioned previously, our study aims were to evaluate veterinary medicine articles that focused on the use of VR or AR, in addition to medical articles that involved animal trials or contained animal data. These human medicine articles could provide us valuable information about the methods performed on animals; therefore, their results could also be useful for veterinarians. After our full-text evaluation, we included 24 articles in our systematic review.

## 3. Results

### 3.1. Literature Overview

Our research strategy was focused on veterinary papers; thus, various medical papers that used AR or VR technology were not included in our study. Based on the previously mentioned research strategy, we selected 24 articles from online databases. The selected articles were published between 2002 and 2022. Table 1 shows the list of our included articles.

### 3.2. Geographical Contribution

**Figure 2:**
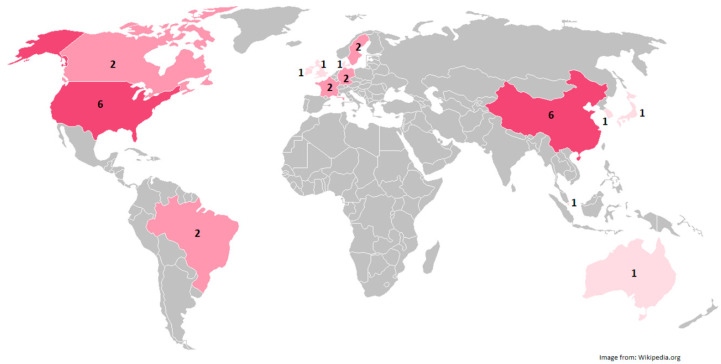
Number of published articles by each country. Four of the included articles were inter-institutional collaborations [34,36,38,49]. We counted these articles considering all collaborating countries; therefore, the sum of the articles is more than 24 in Figure 2.

Most of the included articles were from the USA, China, and European countries. Figure 2 illustrates the number of articles by country.

### 3.3. Topics of Included Articles

**Figure 3:**
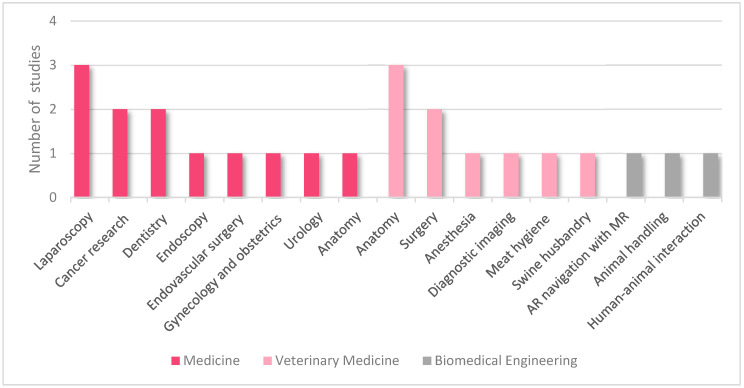
Illustration of included articles per topic. AR: augmented reality; MR: magnetic resonance imaging; US: ultrasonography.

The articles we included in our collection address three main disciplines, including human (n = 12) and veterinary medicine (n = 9), and biomedical engineering (n = 3). The topics in the medical articles consisted of laparoscopy (n = 3), cancer research (n = 2), dentistry (n = 2), endoscopy (n = 1), endovascular surgery (n = 1), gynecology and obstetrics (n = 1), urology (n = 1), and anatomy (n = 1). The veterinary articles discussed veterinary anatomy (n = 3), veterinary clinical science consisting of veterinary surgery, veterinary anesthesia and diagnostic imaging (n = 4), food safety/meat hygiene (n = 1), and swine husbandry (n = 1). Figure 3 shows the representation of the articles per topic.

### 3.4. Annual Distribution of Included Articles

**Figure 4:**
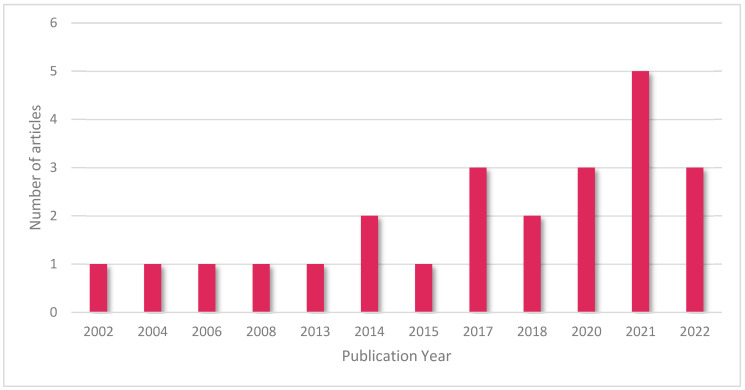
Illustration of number of included articles based on publication year.

The included articles in this study are shown in Figure 4 according to their publication year.

### 3.5. Relevance to Veterinary Medicine

We divided the articles we included in our study into three categories according to their topical relevance to clinical veterinary medicine. We classified articles that used or evaluated the use of VR or AR in veterinary medicine into the first group. Furthermore, we classified articles in human medicine that used VR or AR on animals into the second group. The remaining VR or AR projects using animal models were classified into the third group. Table 2 shows the classified articles.

#### 3.5.1. VR/AR in Veterinary Medicine

All of the articles we included in this category investigated the use of VR or AR in veterinary medicine. From nine included articles, four articles evaluated the use of VR and AR in clinical fields, such as surgery, gynecology and obstetrics, and anesthesia [3,40,48,50]. Three articles were related to veterinary anatomy [38,47,51], and two articles evaluated the use of new technologies, such as VR and AR, to improve the quality of public health and animal welfare [33,46].

In 2013, Lee et al. [40] developed a training simulator for intravenous (IV) injection using AR. The aim of this project was to train veterinary students before they start to perform canine venipuncture on real animals. This simulator was based on a 3D silicone model of a dog arm, which was created from CT scans (DICOM images); an individualized syringe with a gyroscope, vibrator, motor, and light-emitting diode; and AR markers on the model and syringe. The cameras were used to recognize the markers on the model and syringe. The AR simulation including 3D vessels, and the syringe position on the silicon model was projected on a monitor—no HMD was used. The students were divided into an AR-trained group and a control group (without AR training) and both groups were asked to perform injections in live dogs and thereafter complete a questionnaire. The results demonstrated significantly more proficiency for the AR-trained group in this study (*p* ≤ 0.01) [40].

In a case study, Wilkie et al. (2020) [50] used mixed reality to perform a femoral nerve block in dogs. This study aimed to develop a 3D canine leg model without the use of medical images (CT scan or magnetic resonance imaging (MRI)), develop a mixed-reality application, and report the workflow. In this study, a canine cadaver was dissected to expose the femoral nerve and different pictures were taken. These pictures were used to create a 3D model by different tools; furthermore, an AR application was developed. Microsoft HoloLens (Microsoft, Redmond, WA, USA) HMD was used as an AR navigator in this study, and the 3D model was superimposed on the real leg (cadaver). The authors reported a successful workflow despite some limitations in this method. They reported that the use and development of new technologies, such as mixed reality (AR), could help anesthesiologists to perform better nerve blocks by locating the nerves more accurately. 

Hunt et al. (2020) [3] studied a group of pre-graduate veterinary students that watched stereoscopic surgical videos with a VR application and headset before performing ovariohysterectomy surgery on real animals. Their study aimed to investigate whether the preoperative VR simulation can improve the students’ performance in ovariohysterectomy surgery on dogs. This project used a minimally interactive veterinary surgical training VR application (Exero Vet, Pyxis, New Orleans, LA, USA). Furthermore, smartphones and stereoscopic VR headsets (Voxkin, Kathmandu, Nepal) were used as HMDs in this study. Despite the high endorsement rate (83%) from students for this method, no significant difference was recorded between this group and the control group regarding surgical performance scores and time on real animals.

In 2022, Shimada et al. [48], presented a prototype of an AR system that was designed to superimpose 3D CT scans on real animals in the field of view. The study’s aim was to report the methodology of creating a 3D model, technical aspects, and system configurations. The clinical significance of this project was its ability to project and superimpose the 3D CT scans of the patient during surgery in the field of view. Microsoft HoloLens 2 (Microsoft, USA) was also used in this study as an HMD.

The following three articles in this category were related to veterinary anatomy. In 2017, Seo et al. [47] designed a piece of pedagogic VR software with which veterinary students could learn and practice canine anatomy. The aim of this simulator was to provide a new and flexible platform for education of the canine anatomy (canine skeletal system). This simulator was developed with the Unity3D game engine (Unity Technologies, San Francisco, CA, USA) and consisted of an HTC Vive (High Tech Computer Corporation, Taiwan) HMD, two infrared tracking stations, and two motion controllers. Most of the participating students (90.9%) in this study gave positive feedback for the program’s educational applications. One year later, in a similar educational pilot study, Xu et al. [51] used the same HMD and controllers (HTC Vive VR) and developed a VR system from the CT scans of dogs to teach and examine the anatomy knowledge of veterinary students. They used DICOM data to create a 3D dog model. Furthermore, a VR environment was developed by a game engine. After the creation of the VR environment, different functions were implemented in this environment, such as multiple-choice tests, and students used this technology to assess their anatomy knowledge. The majority of the students mentioned that the 3D model makes the test easier and gave positive feedback. In the same year, Christ et al. [38] developed a user-friendly AR application for the education of canine head anatomy and reported the workflow. The authors used canine head CT scans and MRI as input data and developed 3D models by using different tools. Furthermore, an interactive AR application was created. A PC (HP Z230 Workstation), an HTC One M8 mobile phone (High Tech Computer Corporation, Taiwan), and a Samsung Tablet Galaxy 10 were used in this study. The 3D models were imported to these Android mobile phones and tablets to demonstrate the AR. The authors noted that this proof of concept provides a great framework for the development of AR products.

Two of the included articles in this category were related to public health and animal welfare. In 2021, Almqvist et al. [33] assessed the possibility of post-mortem meat inspections in pigs via AR and live-streamed video with the help of a camera-equipped technician. In this study, a live video stream was used to perform remote meat inspection via a technician on-site. The equipment used in this project for on-site technicians consisted of a Samsung Galaxy S9+ smartphone (Samsung Inc. Seoul, Republic of Korea) with Android Version 8.0.0. (Alphabet Inc., Mountain View, CA, USA) and headset. The acquired data were sent to a veterinarian via an internet connection and remote meat inspection was performed with the help of the technician on site. The equipment used in the receiving terminal consisted of a PC with Windows 10 (Microsoft Corp., Redmond, WA, USA). Furthermore, XMReality Remote Guidance software (XMReality AB, Linköping, Sweden) was used for both Android and Windows systems. The result of the remote meat inspection was compared with the on-site inspection. Despite difficulties in translating all of the results into real-world applications, they recorded good-to-high agreements for this method, which was reported as an alternative to traditional meat inspections. In another study, Schütz et al. [46] reported a method for increasing animal welfare and transparency in animal husbandry. The authors held virtual farm tours using Oculus Go (Meta Platforms, Inc., Menlo Park, CA, USA) VR headsets and Samsung Galaxy Tab 3 tablets (Samsung Inc. Seoul, Republic of Korea) that utilized 360-degree videos to assess the differences between these two gadgets and investigate the applicability of using visual farm tours to promote transparency in pig husbandry. Participants were asked to describe their opinions about pig farming after taking a farm tour. Their findings indicate that virtual farm tours could improve transparency in animal husbandry. However, both devices were reported to be practical. The VR headset was reported to be more realistic, while the tablet was reported to be more user-friendly.

#### 3.5.2. VR/AR in Human Medicine Using Animal Models

All of the articles we included in this category are human medicine studies with animal trials or models. Three articles investigated the use of AR/VR in laparoscopic surgeries [32,34,44]. Araujo et al. (2014) [34] investigated the impact of short-duration VR training on the performance of a real laparoscopic colectomy on a porcine model. They used LAP Mentor (Simbionix, Cleveland, OH, USA) to simulate the laparoscopic sigmoid resection. This simulator offers various programs for hands-on laparoscopic training. The authors reported that participants who exercised the laparoscopic colectomy prior to the surgery on the VR simulator had better performance on the real porcine model. Luo et al. (2020) [44] used AR to navigate liver resection on ex vivo porcine liver and in vivo porcine models. This study aimed to develop an AR-assisted method for laparoscopic liver resection and assess the accuracy of these methods. This navigation system consisted of hand–eye calibration, preoperative image segmentation, intraoperative surface reconstruction, image-to-patient registration, and AR navigation [44]. A rigid stereo laparoscope was used to take images for 3D reconstruction in this project; however, a positional tracker was used to acquire the position and orientation of the laparoscope. A monitor with polarized 3D glasses was used to visualize the images [44]. An artificial tumor was injected into a pig under general anesthesia using the guidance of ultrasound in this study. A preoperative CT was performed and copper nails were implemented under the laparoscopic image; then, an intraoperative CT scan was performed and a 3D model was reconstructed. Thereafter, an intraoperative liver surface reconstruction and AR visualization were performed [44]. The results attained from this study confirmed the validity of both ex and in vivo experiments and established that AR-assisted laparoscopic liver resection can be useful in practice. In another laparoscopic study, Adballah et al. (2021) [32] investigated the use of AR-assisted laparoscopic liver resection on ex vivo sheep livers with AR software (Hepataug) and compared the results with standard ultrasonography. In this study, pseudo-tumors (from 10 to 20 mm) were created in sheep cadaveric livers by injecting alginate and CT scans were performed to reconstruct 3D models. The cadaveric livers were placed in a pelvi-trainer and laparoscopic resections were performed with Hepataug software (AR software for hepatic laparoscopy) [32] using standard ultrasound, AR, and a combination of both of these methods. The aim was to obtain free resection margins as close as 1 cm. They reported that AR-assisted laparoscopy with this system provides precise resection margins, which could reduce surgical errors.

Two studies investigated the use of AR-assisted pulmonary nodule localization in animal models. Li et al. (2021) [43] presented a method for the localization of the artificially created pulmonary nodules in canine models with the use of AR navigation. The artificial tumors were created under general anesthesia on canine models and CT images were taken. The acquired data were analyzed and transferred to an AR HMD (Microsoft HoloLens, Microsoft, Washington, DC, USA); thereafter, the operator located these tumors with the help of AR-navigation-guided puncture. Furthermore, all markers were later removed by video-assisted wedge resection or lobectomy. The authors reported that this method was safe and recorded no severe postoperative complications. In a similar study, Peng et al. (2021) [45] used AR for the localization of solitary pulmonary nodules in porcine models and reported the workflow and feasibility of this method in an animal model. They used CT scans (DICOM images) and various software to create a 3D model. This study’s first phase was carried out on a plastic thoracic model, while the second phase was performed on real pigs. They also used Microsoft HoloLens HMD. The authors proposed that this method might be used in the future for surgical treatment of early-stage lung cancer; however, further clinical investigations are still needed [45].

In 2015, Ioannou et al. [39] compared the oral surgery task performance with either a VR surgical simulator or an animal model (ovine jaw). They used VR technology to assess the effects of VR simulation in real-world oral surgery. This simulation was based on a 3D jaw model, which was previously developed from the open-source Forssim library [54], stereo VR glasses, and a sensible Phantom 1.5 High Force haptic device. The authors reported that VR simulations reduced the unnecessary movements of the trainees; however, no significant differences were recorded between the two groups regarding outcome scores. Zhou et al. [53] performed robot-assisted surgery with the help of AR to accomplish mandibular angle split osteotomy on canine models. Due to the high risk of nerve damage, infection, and fractures, this surgery was reported to be an invasive and high-risk procedure requiring a clinically experienced surgeon. This study aimed to develop a new AR-navigated method, evaluate the performance of novice surgeons and assess the accuracy and safety of this method on animal models [53]. They used CT scans to develop 3D models, and an nVisor ST60 (NVIS Company, Reston, VA, USA) HMD for AR navigation. In this study, osteotomy planes were performed on mandibles, and tunnels were drilled in the dogs with the help of AR navigation and robotic assistance. The results showed that, despite some limitations, such as not being accurate enough for more complicated surgeries, being a new method for surgeons (who had a lack of experience in using these devices), and having a low sample size (for both surgeons and animals), a combination of robot-assisted surgery and AR navigation could be safe and useful for novice surgeons [53].

Cassidy et al. (2022) [37] studied the effect of VR training on real endoscopic surgical outcomes in the porcine model. They used a virtual reality endoscopic simulator (GI Mentor; 3D Systems, Rock Hill, SC, USA) in their study on colonoscopy training. Participants were divided into proficiency- and repetition-based groups and were asked to perform endoscopy and colonoscopy on live pigs after receiving VR training. The proficiency-based groups performed the tasks with expert-level benchmarks, whereas the repetition-based group had to complete the tasks for a set number of 10 without considering the quality of the surgery or benchmark. The VR training improved surgical skills in both groups and the proficiency curriculum was reported to be a less time-consuming method. No other considerable differences were observed between the two groups. Therefore, the use of VR simulations can help residents achieve the endoscopic skills they need in practice.

Berry et al. [36] investigated the economic aspects of VR training and compared it with animal model training for endovascular procedures on pigs. They used a VIST (Mentice, Gothenburg, Sweden) simulator for endovascular training in their study regarding digital subtraction angiography, balloon angioplasty, stent implantation, and atherectomy. In addition to the scientific/pedagogic benefits of VR training, which can be translated to better clinical performances in human medicine, VR training is reported to be less expensive than animal models.

Bassil et al. (2017) [35] reported a novel learning method that combined VR simulation and animal models for training in operative and diagnostic hysteroscopy. In this study, end-year obstetrics and gynecology residents performed different surgical skills on VR simulators as well as animal specimens (bovine uterus and bladder) after receiving a full-day workshop. They used VitraMed Pelvicsim and HystSim (Zurich, Switzerland) simulators with and without force feedback simulators, respectively, to perform hysteroscopy skills on bovine uteri and bladders. The participants’ theoretical and operative knowledge was assessed before and after training. The residents’ operative knowledge was considerably increased after training; therefore, this method was reported to be an efficient educational method for residents that can be applied in other endoscopic training programs, such as for gastrointestinal endoscopy or cystoscopy.

In 2014, Li et al. [42] reported technical workflow for ultrasound-guided percutaneous renal access in pigs with AR. This study’s goal was to report a new 3D navigation method based on ultrasound in absence of CT or MRI. They used aligned training shapes to build a general 3D statistical kidney model. Using this model, they reconstructed a patient-specific kidney model from sparse ultrasound slices intraoperatively. Real-time needle tracking is added to the ultrasound images after the kidney model has been reconstructed and percutaneous renal puncture is performed under the guidance of augmented ultrasound. This protocol’s validity was confirmed in this study, and the authors argued that using this method might reduce the rates of post-operative complications, such as bleeding in the clinic.

In 2002, Zanchet and Montero [52] created a 3D VR model of a pig liver according to its correlation to the portal and hepatic veins and determined its segmentations as an educational model for medical students, surgeons, and anatomists. In this study, the portal and hepatic veins of 20 livers were injected with a methyl methacrylate solution and the parenchyma of the liver was removed with chloride acid 35%. These models were assessed according to the origin, number, and distribution of the veins, and different photographs were taken. These photographs were used to generate a 3D model by Studio Max 3.0. Software. The authors reported that these 3D models can be used in VR-based medical education. The article does not mention any VR devices or gadgets [52].

#### 3.5.3. Other VR/AR Projects Using Animal Models

The articles we included in this category were selected from a technical point of view, and reported on workflow, available technologies, and the state of the art.

Vogt et al. (2004) [49] developed an AR navigation system for magnetic resonance-guided (MR-guided) intervention, and an MR-guided needle placement was performed on a gel phantom and, thereafter, on a pig model. They discussed different technical aspects of the project, such as the system description and calibration, required hardware, synchronization between the MR scanner and AR system, data processing, and study limitations. They reported this approach as a precise, reliable, and intuitive method for needle placement. In 2006, Lee et al. [41] developed a wearable system based on mixed reality (AR) to improve human–poultry interaction, which was based on the interaction between visual and tactile sensations. The project’s importance stemmed from including tactile sensations in AR, in addition to developing the ability to communicate remotely via an internet connection. In this project, the authors developed a wearable dress for a chicken and a tangible interactive system for humans. First, a 3D live AR simulation of the chicken was projected via an AR headset. This live AR projection of the chicken was recorded with cameras and was in real-time (via an internet connection). To simulate the sense of touching (haptic feedback), an ultrasound transmitter and vibrotactile actuators were placed on the finger of the pet owner, and two ultrasound receivers were placed on the table. The touch event is sent through the Internet connection to the chicken’s dress, which was equipped with vibrotactile actuators to transfer the same touch feeling. The reaction of the chicken to the touching was represented by a physical doll (avatar), thus the owner has physical interaction with the avatar. The researchers discussed the different technical aspects of developing this prototype in this study. Tang et al. (2021) [12] developed a VR simulation system for animal handling to train undergraduate biomedical students. This study was considerable because the researchers designed a VR system in accordance with the 4Rs (replacement, reduction, refinement, and responsibility) moral principles of animal welfare. They developed the virtual animal-holding simulator (ViSi) with VR technology in this study and, as in the authors’ previous study [55], they also used HTC Vive (High Tech Computer Corporation, Taiwan) HMD in this project. They reported the platform to be a reliable training system for undergraduate students.

### 3.6. Methodology

The methods and technologies used in our selected studies can be classified as either VR or AR. Studies that evaluated VR technology can be categorized into three sub-groups, including non-, semi-, and full-immersive VR. However, AR Studies can be divided into studies that used AR technology with an HMD or AR headset, and studies that used AR technology without HMD (with a mobile phone, a screen, or a projector).

Of the articles we included in our review, 11 and 13 studies were based on VR and AR, respectively. Among the VR studies, one of them was based on non-immersive VR and was performed using a monitor or a screen [52], four articles were based on semi-immersive VR [34,35,36,37], and six of them were based on full-immersive VR [3,12,39,46,47,51].

Most of the simulations with semi-immersive VR were performed using one or several monitors and a surface or platform to perform surgical maneuvers such as VIST simulator (Mentice, Gothenburg, Sweden) for endovascular training [36], LAP Mentor (Simbionix, Cleveland, OH, USA) for laparoscopic surgery [34], VitraMed Pelvicsim and HystSim simulators (Zurich, Switzerland) [35] or GI Mentor (3D Systems, Rock Hill, SC, USA) [37]. The most frequently used full-immersive VR gadgets in the articles consisted of HTC Vive (High Tech Computer Corporation, Taiwan) [12,47,51] and Oculus Go (Meta Platforms, Inc., Menlo Park, CA, USA) [46]. However, one of the studies was performed using smartphones and stereoscopic VR headsets (Voxkin, Kathmandu, Nepal) [3].

**Figure 5:**
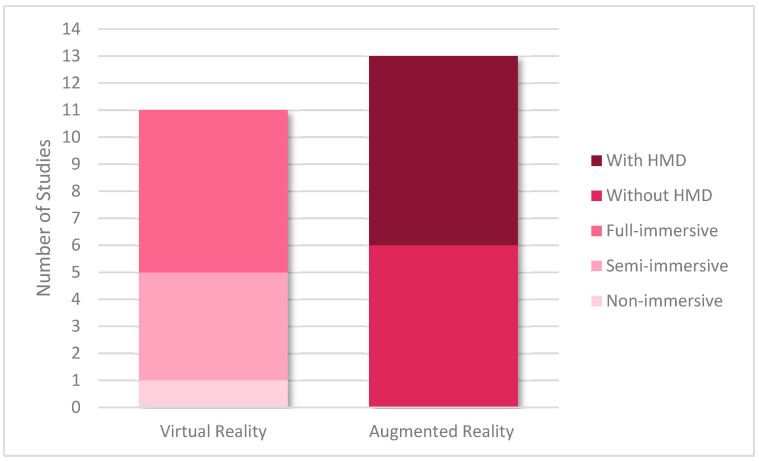
Illustration of included articles based on used technologies. HMD: head-mounted display.

Of the total AR studies, seven were performed with an HMD or headset [41,43,45,48,49,50,53]. Different HMDs were used in AR studies in this category. The most famous and frequently used AR HMDs in included articles consisted of Microsoft HoloLens (Microsoft, Washington, DC, USA) [43,45,48,50] and nVisor ST60 (NVIS, USA) [53]. An HMD is a device that is worn on the head and contains a display that can be mono- or binocular with speakers. Different types of HMD exist, and these headsets have different technical features. The HMDs that are used in AR projects are mostly optical head-mounted displays (OHMD), which reflect projected images on a transparent display that the user can see the real world through. Furthermore, six articles were performed without using HMDs [32,33,38,40,42,44]. Most of the included articles in this category were performed using monitors [32,33,38,40,42,44]. However, in some of the studies, AR simulations were performed using smartphones or tablets [33,38]. We classified our included articles according to whether they used VR or AR technology (Figure 5).

## 4. Discussion

We carried out a systematic review to assess the use of VR and AR in veterinary science. Despite the high number of scientific articles in medical science that used these technologies, few studies have been undertaken in veterinary medicine [3,33,38,40,46,47,48,50,51]. However, these technologies are new in medical science and a high number of studies are still needed to make a strong conclusion about the use of these methods in human and veterinary medicine [32,34,39,40,42,43,44,45,48,50,53]. Furthermore, separate studies have designed methods for each field, and a general conclusion cannot yet be made for all specialties. The possibility of using these technologies in daily therapeutic and diagnostic processes, evaluating the economic aspects of these methods, and comparing them with traditional methods, in addition to assessing the practicability of these methods in real life, are considerable issues that have to be further explored. Because different people with different levels of expertise will use these technologies, intra- and inter-observer studies should be designed to assess the repeatability and reproducibility of the protocols reported in our review, especially when using AR navigation in real-life scenarios, such as laparoscopic interventions, tumor marking, surgical planning, and similar diagnostic tasks. The results of these studies would allow scientists to better understand the pros and cons of each method and propose a solution, thereby accelerating the development of these methods or devices.

Even though these technologies are new, the development process has rapidly advanced over the past few years, with considerable achievements being obtained. Through the rapid and intense growth of science in different fields, especially in medicine, computer, engineering, and communication sciences in recent years, medical science, including human and veterinary medicine, is predicted to greatly benefit from innovations in technologies, such as VR, AR, AI, machine learning, the metaverse, etc. The globalization and expansion of internet connections, upgrades to 5G internet, new hardware and gadgets, and progress in applied sciences, are accelerating the use of these cutting-edge technologies in human and veterinary medicine. However, the COVID-19 pandemic has resulted in new demands on educational platforms and initiatives in academia. Thus, it is necessary to upgrade, transform, and align classic educational methods and curricula with the latest methods and technologies in the post-pandemic era.

Using AR and VR in veterinary medicine will not only increase productivity in pedagogy and clinical skills but provide an appropriate alternative to animal trials to comply with the 3Rs (replacement, reduction, refinement) principle [3,5].

From an economic point of view, using these methods in educational programs is beneficial in comparison with animal trials or training on cadaveric specimens, reducing training costs [36]; however, using these methods on a daily basis as diagnostic/therapeutic tools may not be economic for small clinics yet because of the high costs of the equipment and the need to hire specialists to operate it, as well as its time-consuming nature. At the same time, increasing the supply and demand of these products would decrease the price and increase the future quality of products and services.

Generally, the VR/AR articles we included in our review can be divided into studies that focus on pedagogy and undergraduate education, such as those developing new platforms for education regarding the anatomy [38,47,51], animal handling training [12], or venipuncture training [40] and studies that focus on postgraduate education or professional/clinical training, such as those that provide laparoscopic simulations [32,34,37,44] or dentistry training [39,53], or report new or alternative diagnostic/therapeutic methods [42,43,45]. These articles explored methods for the use of VR or AR in medical science. Full-immersive VR and AR may be considered state of the art in these fields as a result of these technologies transferring a wide range of 3D visual, auditory, and even haptic information.

Technical support for these technologies is another considerable topic to be discussed regarding the use of AR/VR in veterinary medicine. Based on the nature and aims of the studies, various technical procedures should be performed to ensure the operability of these systems. Furthermore, engineers, experts, and technicians are required for this purpose (research, design, and development), in addition to various software and numerous programs that are, in many cases, not free. Therefore, we propose these issues as the limitations of these technologies. One of the most considerable challenges in the daily use of these tools is the lack of a standard methodology for the use and processing of raw data, such as DICOM data. As previously reported, the raw input data should be processed and analyzed according to the desired outcome, which can be a time-consuming procedure. However, the standardization of these procedures can facilitate the application of these technologies in both academia and practice. In addition, new start-ups can be established to fill the gaps between manufacturing companies and academia or clinics by providing technological services.

Finally, the calibration of the devices, as well as the low sample sizes that characterize our included articles, are additional study limitations. System calibration, especially for AR projects where a real-time projection of the virtual structures has to be performed, is a considerable factor because improper calibration can increase bias. Furthermore, most of the clinical studies we included in our review had a limited sample size. Therefore, we recommend increases in sample sizes so that future studies can arrive at firm conclusions. Ultimately, VR and AR technologies can be considered supporting tools in veterinary medicine, and with their advancement, they may even become potential alternatives in some diagnostic fields in the future.

## 5. Conclusions

VR and AR are rapidly developing cutting-edge technologies that are gaining attention in veterinary medicine. In this systematic review, we aimed to evaluate studies for their use of VR/AR in veterinary medicine, as well as human medicine with animal trials. Twenty-four articles were included in this systematic review by screening the Scopus, PubMed, and Web of Science databases. In some cases, these technologies may offer an alternative to animal trials or invasive interventions, according to the studies. Furthermore, using these tools alongside classic educational programs and professional training can increase the scientific output of these courses and reduce economic costs. Moreover, defining standard protocols for using VR and AR in veterinary medicine can accelerate improvements in the designed systems, make the output of scientific articles more comparable, and help scientists to troubleshoot the designed systems. Finally, we are optimistic regarding the future of virtual healthcare and expect considerable innovations and developments; therefore, the application of AR/VR can unlock new frontiers in the diagnostic, therapeutic, and pedagogic aspects of veterinary medicine.

## Figures and Tables

**Table 1:** Overview of studies included in systematic review.

Author	Animal Model	Discipline	Used Technology	Type of Study	Aim of Study
Adballah et al. 2021 [32]	Sheep liver	Medicine (laparoscopy)	Non-HMD-based AR	Randomized controlled trial	Test the performance of AR software in tumor resection
Almqvist et al. 2021 [33]	Pig	Food Safety (meat hygiene)	Non-HMD-based AR	Non-randomized clinical trial	Remote post-mortem meat inspection in pigs
Araujo et al. 2014 [34]	Swine	Medicine (laparoscopy)	Semi-immersive VR	Randomized controlled trial	Assess the effect of colectomy training with VR
Bassil et al. 2017 [35]	Bovine	Gynecology and Obstetrics	Semi-immersive VR	Before-and-after	Assess the combination of animal models and VR simulations
Berry et al. 2008 [36]	Swine	Medicine (endovascular surgery)	Semi-immersive VR	Case-control	Assess economic aspects of the VR simulation in endovascular surgery training
Cassidy et al. 2022 [37]	Swine	Medicine (endoscopy)	Semi-immersive VR	Randomized controlled trial	Compare proficiency-based vs. repetition-based VR training in swine colonoscopy
Christ et al. 2018 [38]	Canine	Veterinary Medicine (anatomy)	Non-HMD-based AR	Proof of concept	Develop a canine head anatomic model
Hunt et al. 2020 [3]	Canine	Veterinary Medicine (surgery)	Full-immersive VR	Randomized controlled trial	Effect of VR simulation on first surgical performance
Ioannou et al. 2015 [39]	Ovine	Dentistry	Full-immersive VR	Randomized controlled trial	Effect of VR simulation on surgical performance
Lee et al. 2013 [40]	Canine	Veterinary Medicine	Non-HMD-based AR	Randomized controlled trial	AR intravenous injection simulator for veterinary students
Lee et al. 2006 [41]	Poultry	Computer Science (human–poultry interaction)	HMD-based AR	Proof of concept	Developing a mixed-reality system for human–animal interactions
Li et al. 2014 [42]	Swine	Biomedical Engineering	Non-HMD-based AR	Non-randomized clinical trial	Augmented-ultrasound-guided renal puncture in pigs
Li et al. 2021 [43]	Canine	Medicine (Thorax Surgery)	HMD-based AR	Non-randomized clinical trial	AR navigation-guided pulmonary nodule localization in dogs
Luo et al. 2020 [44]	Swine	Medicine (Laparoscopy)	Non-HMD-based AR	Qualitative and quantitative reporting results on usability	AR-assisted navigation system for laparoscopic liver resection in pigs
Peng et al. 2021 [45]	Swine	Medicine (Thorax Surgery)	HMD-based AR	Non-randomized clinical trial	AR-assisted localization of pulmonary nodules in pigs
Schütz et al. 2022 [46]	Swine husbandry	Veterinary Medicine (Swine Husbandry)	Full-immersive VR	Pilot	Using virtual farm tours to provide insights into pig farms
Seo et al. 2017 [47]	Canine	Veterinary Medicine (Anatomy)	Full-immersive VR	Pilot	Defining learning methods in the VR canine skeletal system
Shimada et al. 2022 [48]	Animal	Veterinary Medicine (Anatomy/Surgery)	HMD-based AR	Prototype testing	Prototype of an AR system to support animal surgery
Tang et al. 2021 [12]	Virtual mouse	Biomedical Science	Full-immersive VR	Pilot	Using VR simulation for animal handling
Vogt et al. 2004 [49]	Swine	Medicine/ Biomedical Engineering	HMD-based AR	Pilot	AR navigation system for MR-guided needle placement procedure in pigs
Wilkie et al. 2020 [50]	Canine	Veterinary Medicine (Anesthesia)	HMD-based AR	Pilot	AR-guided femoral nerve block in a dog
Xu et al. 2018 [51]	Canine	Veterinary Medicine (Anatomy)	Full-immersive VR	Pilot	Creating a VR application for teaching and examining dog anatomy in veterinary education
Zanchet and Montero 2002 [52]	Pig Liver	Medicine (Anatomy)	Non-immersive VR	Proof of concept	Developing a VR model for the evaluation of pig liver anatomy
Zhou et al. 2017 [53]	Canine	Dentistry	HMD-based AR	Non-randomized clinical trial	Robot-assisted mandibular drilling in the canine mandible using AR

HMD: head-mounted display; AR: augmented reality; VR: virtual reality; MR: magnetic resonance.

**Table 2:** Classification of included articles based on topics.

VR/AR in Veterinary Medicine	VR/AR in Human Medicine Using Animal Models	Other VR/AR Projects Using Animal Models
Almqvist et al. 2021 [33]	Adballah et al. 2021 [32]	Lee et al. 2006 [41]
Christ et al. 2018 [38]	Araujo et al. 2014 [34]	Tang et al. 2021 [12]
Hunt et al. 2020 [3]	Bassil et al. 2017 [35]	Vogt et al. 2004 [49]
Lee et al. 2013 [40]	Berry et al. 2008 [36]	
Schütz et al. 2022 [46]	Cassidy et al. 2022 [37]	
Seo et al. 2017 [47]	Ioannou et al. 2015 [39]	
Shimada et al. 2022 [48]	Li et al. 2014 [42]	
Wilkie et al. 2020 [50]	Li et al. 2021 [43]	
Xu et al. 2018 [51]	Luo et al. 2020 [44]	
	Peng et al. 2021 [45]	
	Zanchet and Montero 2002 [52]	
	Zhou et al. 2017 [53]	

## Data Availability

Not applicable.
